# Models with indirect genetic effects depending on group sizes: a simulation study assessing the precision of the estimates of the dilution parameter

**DOI:** 10.1186/s12711-019-0466-6

**Published:** 2019-05-30

**Authors:** Marzieh Heidaritabar, Piter Bijma, Luc Janss, Chiara Bortoluzzi, Hanne M. Nielsen, Per Madsen, Birgitte Ask, Ole F. Christensen

**Affiliations:** 1 Department of Molecular Biology and Genetics, Center for Quantitative Genetics and Genomics, Aarhus University, Tjele, Denmark; 2 Animal Breeding and Genomics Centre, Wageningen University, Wageningen, The Netherlands; 3 Present Address: SEGES, Danish Pig Research Centre, Copenhagen, Denmark

## Abstract

**Background:**

In settings with social interactions, the phenotype of an individual is affected by the direct genetic effect (DGE) of the individual itself and by indirect genetic effects (IGE) of its group mates. In the presence of IGE, heritable variance and response to selection depend on size of the interaction group (group size), which can be modelled via a ‘dilution’ parameter (*d*) that measures the magnitude of IGE as a function of group size. However, little is known about the estimability of *d* and the precision of its estimate. Our aim was to investigate how precisely *d* can be estimated and what determines this precision.

**Methods:**

We simulated data with different group sizes and estimated *d* using a mixed model that included IGE and *d*. Schemes included various average group sizes (4, 6, and 8), variation in group size (coefficient of variation (*CV*) ranging from 0.125 to 1.010), and three values of *d* (0, 0.5, and 1). A design in which individuals were randomly allocated to groups was used for all schemes and a design with two families per group was used for some schemes. Parameters were estimated using restricted maximum likelihood (REML). Bias and precision of estimates were used to assess their statistical quality.

**Results:**

The dilution parameter of IGE can be estimated for simulated data with variation in group size. For all schemes, the length of confidence intervals ranged from 0.114 to 0.927 for *d*, from 0.149 to 0.198 for variance of DGE, from 0.011 to 0.086 for variance of IGE, and from 0.310 to 0.557 for genetic correlation between DGE and IGE. To estimate *d*, schemes with groups composed of two families performed slightly better than schemes with randomly composed groups.

**Conclusions:**

Dilution of IGE was estimable, and in general its estimation was more precise when *CV* of group size was larger. All estimated parameters were unbiased. Estimation of dilution of IGE allows the contribution of direct and indirect variance components to heritable variance to be quantified in relation to group size and, thus, it could improve prediction of the expected response to selection in environments with group sizes that differ from the average size.

**Electronic supplementary material:**

The online version of this article (10.1186/s12711-019-0466-6) contains supplementary material, which is available to authorized users.

## Introduction

Most livestock species are housed in groups in which individuals interact socially and can influence each other’s phenotype. Thus, from a genetics perspective, the phenotype of an individual is influenced by the direct genetic effect (DGE) of the individual itself and by the indirect genetic effects (IGE) of the other individuals (group mates) [1–3]. Theory-based research has demonstrated that IGE affect the rate and direction of response to selection [1, 4]. Furthermore, in the presence of IGE, heritable variance and response to selection depend on the number of individuals that interact (referred to as group size) [1, 4]. The dependency of the magnitude of IGE on group size [5–7] has been modelled using a function of group size and a ‘dilution’ parameter (*d*) [6, 7]. Estimation of *d* is particularly important for species for which the sizes of the groups vary fundamentally and for traits that are recorded over time (such as gain, feed efficiency, or longevity), for which group size may change over time. For instance, in layer chickens, the average group size can vary from 5 to 40 [8, 9]. For layer breeding programs, group size will remain constant over time, apart from mortality. However, in pig breeding (with an average group size of 8 to 15 [10]), group size can vary more because barn and pen sizes, both within and between farms, depend on e.g. choices of the farmer and economic factors. In such a situation, animals from the same genetic line appear in a mix of group sizes within and across farms and, thus, it is necessary to investigate the relationship between IGE and group size for proper estimation of variance components, including for IGE, and consequently for proper interpretation of response to selection in a breeding program. Thus, when group size varies, a statistical model that takes the relationship between the magnitude of IGE and group size into account is required [6, 7].

Three statistical models have been proposed to model the relationship between IGE and group size [6, 7, 11]. In the current study, we used the model of Bijma [6] because it is easier to implement and interpret, since it involves only one parameter for the degree of dilution, while the model proposed by Hadfield and Wilson [7] involves the estimation of additional covariance parameters. Moreover, the model developed by Anacleto et al. [11], which is a non-linear IGE model and uses adaptive Bayesian computational techniques to estimate the model parameters, is more suitable for modelling infectious diseases [11].

In the model of Bijma [6], the dilution parameter *d* can range from 0 to 1 in its Eq. 3: $a_{{{\text{I}}_{i,n} }} = \frac{{a_{{{\text{I}}_{i,2} }} }}{{\left( {n - 1} \right)^{d} }}$, where $a_{{{\text{I}}_{i,n} }}$ is the IGE of individual *i* in a group of *n* members, and $a_{{{\text{I}}_{i,2} }}$ is the IGE of *i* in a group of two members. When there is no dilution (*d* = 0), IGE are independent of group size and when *d* = 1 (full dilution), IGE are inversely proportional to the size of the group. Generally, the magnitude of *d* can be trait- and population-specific [6]. Ignoring *d* results in the overestimation of both the total heritable variance $(\sigma_{\text{TBV}}^{2} )$, which is equal to $\sigma_{{a_{\text{D}} }}^{2} + 2\left( {n - 1} \right)\sigma_{{a_{\text{DI}} }} + \left( {n - 1} \right)^{2} \sigma_{{a_{\text{I}} }}^{2}$, (see Table 1 for a notation key), and of the potential of a population to respond to selection in larger groups [6, 12]. With dilution (*d*), the total heritable variance is: $\sigma_{{a_{\text{D}} }}^{2} + 2\left( {n - 1} \right)^{1 - d} \sigma_{{a_{\text{DI}} }} + \left( {n - 1} \right)^{2 - 2d} \sigma_{{a_{\text{I}} }}^{2}$ [6]. With incomplete dilution (*d* < 1), the total heritable variance increases with group size, while with complete dilution (*d* = 1), the total genetic variance does not depend on group size and will, therefore, be the same for all group sizes [6].

**Table 1:** Notation of parameters and effects

Symbol	Meaning
*i*, *j*	Focal individual, group mate of focal individual
*P*_*i*_	Phenotype of individual *i*
DGE, IGE	Direct genetic effect, indirect genetic effect
*T*, *n*_g_, *n*, $\bar{n}$	Total number of individuals, number of groups, group size, average group size
$P_{{{\text{D}}_{i} }} ,P_{{{\text{I}}_{i} }}$	Direct phenotypic effect of *i*, indirect effect of *i*
$a_{{{\text{D}}_{i} }} ,a_{{{\text{I}}_{i} }}$	Direct genetic effect of *i*, indirect genetic effect of *i*
$E_{{{\text{D}}_{i} }} ,E_{{{\text{I}}_{i} }}$	Direct non-genetic effect of *i*, indirect non-genetic effect of *i*
$\sigma_{{a_{\text{D}} }}^{2} ,\sigma_{{a_{\text{I}} }}^{2}$	Variance of DGE among individuals, variance of IGE among individuals
$\sigma_{{E_{\text{D}} }}^{2} ,\sigma_{{E_{\text{I}} }}^{2}$	Direct non-genetic variance, indirect non-genetic variance
$\sigma_{{a_{\text{DI}} }} ,r_{{a_{\text{DI}} }}$	Covariance between DGE and IGE, genetic correlation between direct and indirect effects
$\sigma_{{E_{\text{DI}} }} ,r_{{E_{\text{DI}} }}$	Non-genetic direct–indirect covariance, non-genetic correlation between direct and indirect effects
$\sigma_{{P_{\text{D}} }}^{2} ,\sigma_{{P_{\text{I}} }}^{2}$	Direct phenotypic variance, indirect phenotypic variance
$\sigma_{\text{TBV}}^{2}$	Total heritable variance
$h_{\text{D}}^{2} ,h_{\text{I}}^{2}$	Direct heritability, indirect heritability
SE	Standard error
*d*	Dilution
*CV*	Coefficient of variation

Several studies have investigated estimation of IGE [12–19] and the contribution of IGE to heritable variance, either in real or simulated data with a constant group size (see review by Bijma [20]). However, knowledge about the impact of varying group sizes on estimability of genetic parameters and the dilution parameter (*d*) is limited.

Here, we used the model proposed by Bijma [6] to simulate data with varying group sizes and to estimate *d* and other parameters in the model such as the genetic variances of DGE and IGE and the genetic correlation between DGE and IGE. To investigate how precisely *d* can be estimated and what determines this precision, we used simulated schemes that differed in variability of group-size, quantified by the coefficient of variation (*CV*), and in average group size. Two designs for allocation of individuals to groups were tested: (1) a random design and (2) a two-family design. For the random design, individuals were randomly allocated to groups. The two-family design, in which each group was composed of two families, was used to investigate if it yielded more precise estimates of *d* than the random design, as was previously shown for estimates of the variance of IGE with fixed group sizes [21, 22]. In addition, we hypothesized that estimates would be more precise for schemes with larger *CV* of group size, since the impact of *d* on the phenotype is larger with larger *CV* of group size (see “Methods” section).

## Methods

### Simulation

A population with two discrete generations was simulated using R [23]. The base population included 50 sires and 200 dams all unrelated. To generate the second generation, sires and dams from the base population were mated at random. Each sire was mated to four dams and each dam produced 40 full-sib offspring, resulting in 8000 simulated individuals. Both direct and indirect effects had a genetic and a non-genetic component. The sex of each individual was randomly assigned with equal probability. The DGE and IGE of each individual in the base population were sampled from a bivariate normal distribution: $$BVN\left( {\left[ {\begin{array}{*{20}c} 0 \\ 0 \\ \end{array} } \right],\left[ {\begin{array}{*{20}c} {\sigma_{{a_{\text{D}} }}^{2} } & {\sigma_{{a_{\text{DI}} }} } \\ {\sigma_{{a_{\text{DI}} }} } & {\sigma_{{a_{\text{I}} }}^{2} } \\ \end{array} } \right]} \right)$$ (see Table 1 for a notation of parameters and effects). DGE and IGE of the individuals in the second generation were calculated as: $a_{\text{D}} = \frac{1}{2}a_{{{\text{D}}_{\text{sire}} }} + \frac{1}{2}a_{{{\text{D}}_{\text{dam}} }} + MS_{\text{D}}$ and $a_{\text{I}} = \frac{1}{2}a_{{{\text{I}}_{\text{sire}} }} + \frac{1}{2}a_{{{\text{I}}_{\text{dam}} }} + MS_{\text{I}}$, where $a_{{{\text{D}}_{\text{sire}} }}$ and $a_{{{\text{I}}_{\text{sire}} }}$ are the DGE and IGE of the sire, $a_{{{\text{D}}_{\text{dam}} }}$ and $a_{{{\text{I}}_{\text{dam}} }}$ are the DGE and IGE of the dam, and $MS_{\text{D}}$ and $MS_{\text{S}}$ are the direct and indirect Mendelian sampling components, which were sampled from $$BVN\left( {\begin{array}{*{20}l} 0 \hfill \\ 0 \hfill \\ \end{array} ,\frac{1}{2}\left[ {\begin{array}{*{20}l} {\sigma_{{a_{\text{D}} }}^{2} } \hfill & {\sigma_{{a_{\text{DI}} }} } \hfill \\ {\sigma_{{a_{\text{DI}} }} } \hfill & {\sigma_{{a_{\text{I}} }}^{2} } \hfill \\ \end{array} } \right]} \right)$$. Direct and indirect non-genetic components were similarly sampled from $$BVN\left( {\left[ {\begin{array}{*{20}c} 0 \\ 0 \\ \end{array} } \right],\left[ {\begin{array}{*{20}c} {\sigma_{{E_{\text{D}} }}^{2} } & {\sigma_{{E_{\text{DI}} }} } \\ {\sigma_{{E_{\text{DI}} }} } & {\sigma_{{E_{\text{I}} }}^{2} } \\ \end{array} } \right]} \right)$$. Both generations were included in the pedigree but phenotypic values were only generated for the second generation.

Bijma [6] proposed to model the dilution of IGE as: $a_{{{\text{I}}_{i,n} }} = \frac{{a_{{{\text{I}}_{i,2} }} }}{{\left( {n - 1} \right)^{d} }}$, where $a_{{{\text{I}}_{i,n} }}$ is the IGE of individual *i* in a group of *n* members, $a_{{{\text{I}}_{i,2} }}$ is the IGE of *i* in a group of two members, and d is the degree of dilution. When d = 0, IGE does not depend on group size, and when d = 1, IGE is inversely proportional to the number of group mates [6]. The degree of *d* can be estimated from data with varying group size and IGE can be estimated as a function of average group size $(\bar{n})$ as: $a_{{{\text{I}},\bar{n}}} = \left( {\frac{{\bar{n} - 1}}{n - 1}} \right)^{d} a_{\text{I}}$ [6]. As explained in the next paragraph, in the simulation, both indirect genetic and non-genetic effects were scaled by $\left( {\frac{{\bar{n} - 1}}{n - 1}} \right)^{d}$.

The phenotype of each individual in the second generation consisted of a direct effect $(P_{{{\text{D}}_{i} }} )$ and the sum of the indirect effects $(P_{{{\text{I}}_{j} }} )$ of each of its group mates [1]. Finally, the phenotype of each individual used for subsequent estimation of variance components was computed by scaling the indirect genetic and non-genetic effects depending on group size and summing all effects as follows:

1 $$P_{i} = a_{{{\text{D}}_{i} }} + \left( {\frac{{\bar{n} - 1}}{n - 1}} \right)^{d} \mathop \sum \limits_{j \ne i}^{n - 1} a_{{{\text{I}}_{j} }} + E_{{{\text{D}}_{i} }} + \left( {\frac{{\bar{n} - 1}}{n - 1}} \right)^{d} \mathop \sum \limits_{j \ne i}^{n - 1} E_{{{\text{I}}_{j} }} ,$$

where $\left( {\frac{{\bar{n} - 1}}{n - 1}} \right)^{d} \sum\nolimits_{j \ne i}^{n - 1} {a_{{{\text{I}}_{j} }} }$ and $\left( {\frac{{\bar{n} - 1}}{n - 1}} \right)^{d} \sum\nolimits_{j \ne i}^{n - 1} {E_{{{\text{I}}_{j} }} }$ are the sum of the indirect genetic and indirect non-genetic effects, respectively, over the *n* − 1 group mates (*j*) of the focal individual *i*. Details about parameters values used in the simulation are shown in a section below.

### Simulated schemes

In total, 18 schemes were simulated (Table 2) with different average group sizes (4, 6, and 8) and variation in group size (*CV* = coefficient of variation, ranging from 0.125 to 1.010). For investigating the estimability of *d*, group size must vary because *d* is irrelevant if there is no variation in group size. We chose *CV* as the measure of variation in group size that affects the precision of the estimate of *d* because we hypothesized that *d* can be estimated more precisely if the variance of $\left( {\frac{{\bar{n} - 1}}{n - 1}} \right)^{d}$ is larger, which occurs when *n* varies more relative to its average (i.e. the *CV*).

**Table 2:** Schemes used to simulate data

Scheme^a^	*n*	*n*_*g*_^b^	*CV*	$\bar{n}$
1	3, 4, 5	889, 667, 533	0.250	4
2	3, 5	1335, 799	0.354	4
3	2, 4, 6	1333, 666, 445	0.500	4
4	2, 6	2002, 666	0.707	4
5	5, 6, 7	534, 445, 380	0.167	6
6	5, 7*	799, 572*	0.236	6
7	4, 6, 8	666, 444, 334	0.333	6
8	4, 8	1000, 500	0.471	6
9	2, 6, 10	1333, 444, 267	0.667	6
10	2, 10	2000, 400	0.943	6
11	7, 8, 9**	380, 333, 297**	0.125	8
12	7, 9	572, 444	0.177	8
13	6, 8, 10	446, 333, 266	0.250	8
*14*	6, 10***	666, 400***	*0.353*	*8*
15	4, 8, 12	666, 334, 222	0.500	8
*16*	*4, 12*	*1001, 333*	*0.707*	*8*
17	2, 8, 14	1334, 334, 190	0.750	8
*18*	*2, 14*	*2005, 285*	*1.010*	*8*

For some schemes, a few individuals were removed if they could not make up a group for the group sizes of that scheme

For the schemes in italics (14, 16 and 18), the two-family designs were also tested

*n* group size, *n*_*g*_ number of groups, *CV* coefficient of variation and $\bar{n}$ average group size

*One individual was removed; **three individuals were removed; ***four individuals were removed

^a^For each scheme, three dilution parameters were evaluated

^b^The 8000 individuals were divided into either two or three subsets depending on the number of group sizes in a scheme and the subsets were divided by the group size, resulting in the number of groups

### Parameter values for simulated schemes

For all simulated schemes, three values of *d* (0, 0.5, and 1) were evaluated (Table 3). Parameter values $\sigma_{{a_{\text{I}} }}^{2}$ and $\sigma_{{E_{\text{I}} }}^{2}$ were defined for the average group size, as was proposed in Eq. 7 in Bijma [6], in which $\sigma_{{a_{\text{I}} }}^{2}$ and $\sigma_{{E_{\text{I}} }}^{2}$ were scaled by $\left( {\frac{{\bar{n} - 1}}{n - 1}} \right)^{d}$. For a fair comparison between schemes with different average group sizes but the same value of *d*, the indirect effects for a given group size should be comparable across schemes. In other words, when $d > 0$, the values assigned to $\sigma_{{a_{\text{I}} }}^{2}$ and $\sigma_{{E_{\text{I}} }}^{2}$ were different for schemes that differed in average group size and were calculated using the scaling factor $\left( {\frac{{\bar{n} - 1}}{n - 1}} \right)^{2d}$, which determines the change in total variance due to IGE with a change in group size (an example is shown below). This scaling was applied to avoid having large $\sigma_{{a_{\text{I}} }}^{2}$ and $\sigma_{{E_{\text{I}} }}^{2}$ for schemes with a large average group size. Table 3 lists the values that were assigned to the indirect genetic and non-genetic variances and shows that the scaled variances were the same across schemes that had the same value for *d* but different average group sizes. In other words, the schemes with the same value for *d* are comparable, since the same dilution was applied both between and within the schemes. For example, consider scheme 1 (3, 4, 5) and scheme 7 (4, 6, 8), for $d = 0.5$ (note that both schemes include groups of size 4). Scheme 1 has $\sigma_{{a_{{{\text{I}},\bar{n}}} }}^{2} = 0.1$ for a group of $n = \bar{n} = 4$. Therefore, we chose the value of $\sigma_{{a_{{{\text{I}},\bar{n}}} }}^{2}$ for scheme 7 such that, for $n = 4$, the $\sigma_{{a_{\text{I}} }}^{2}$ is also equal to 0.1. The resulting value for scheme 7 was $\sigma_{{a_{{{\text{I}},\bar{n}}} }}^{2} = 0.06$, such that $\sigma_{{a_{\text{I}} }}^{2} \left( {n = 4} \right) = 0.06*\left( {\frac{6 - 1}{4 - 1}} \right)^{{\left( {2 *0.5} \right)}} = 0.1,$ which is the required value (Table 3) and Additional file 1: Table S1. For each value of *d*, values of $\sigma_{{a_{\text{I}} }}^{2}$ and $\sigma_{{E_{\text{I}} }}^{2}$ for the schemes with $\bar{n} = 4$ were considered to be the base values (Table 3, and Additional file 1: Table S1). For all schemes, $r_{{a_{\text{DI}} }}$ was set to 0.

**Table 3:** Parameter values used for simulation

Parameter	*d* = 0			*d* = 0.5			*d* = 1		
	$\bar{n} = 4$	$\bar{n} = 6$	$\bar{n} = 8$	$\bar{n} = 4$	$\bar{n} = 6$	$\bar{n} = 8$	$\bar{n} = 4$	$\bar{n} = 6$	$\bar{n} = 8$
$\sigma_{{a_{\text{D}} }}^{2}$	0.300	0.300	0.300	0.300	0.300	0.300	0.300	0.300	0.300
$\sigma_{{a_{\text{I}} }}^{2}$	0.100	0.100	0.100	0.100	0.060	0.043	0.100	0.036	0.018
$\sigma_{{E_{\text{D}} }}^{2}$	0.700	0.700	0.700	0.700	0.700	0.700	0.700	0.700	0.700
$\sigma_{{E_{\text{I}} }}^{2}$	0.230	0.230	0.230	0.230	0.138	0.099	0.230	0.083	0.042
$r_{{a_{\text{DI}} }}$	0.000	0.000	0.000	0.000	0.000	0.000	0.000	0.000	0.000
$\sigma_{\text{TBV}}^{2}$	1.200	2.800	5.200	1.200	1.800	2.410	1.200	1.200	1.200
$h_{\text{D}}^{2} = h_{\text{I}}^{2}$	0.300	0.300	0.300	0.300	0.300	0.300	0.300	0.300	0.300
$\sigma_{{P_{\text{D}} }}^{2}$	1.000	1.000	1.000	1.000	1.000	1.000	1.000	1.000	1.000
$\sigma_{{P_{\text{I}} }}^{2}$	0.330	0.330	0.330	0.330	0.198	0.142	0.330	0.119	0.060

Parameter values used for the simulated schemes with three average group sizes $\bar{n} = 4, \, 6,{\text{ or }}8$ and three values of *d* = 0, 0.5, or 1

A moderate heritability (both direct and indirect heritability, $h_{\text{D}}^{2} = h_{\text{I}}^{2} = 0.3$) was used (Table 3). Direct heritability is defined as: $h_{\text{D}}^{2} = \sigma_{{a_{\text{D}} }}^{2} /\sigma_{{P_{\text{D}} }}^{2} = \sigma_{{a_{\text{D}} }}^{2} /\left( {\sigma_{{a_{\text{D}} }}^{2} + \sigma_{{E_{\text{D}} }}^{2} } \right)$ and indirect heritability as: $h_{\text{I}}^{2} = \sigma_{{a_{\text{I}} }}^{2} /\sigma_{{P_{\text{I}} }}^{2} = \sigma_{{a_{\text{I}} }}^{2} /(\sigma_{{a_{\text{I}} }}^{2} + \sigma_{{E_{\text{I}} }}^{2} )$. Direct phenotypic variance $(\sigma_{{P_{\text{D}} }}^{2} )$ was set to 1, resulting in a direct genetic variance of $\sigma_{{a_{\text{D}} }}^{2} = h_{\text{D}}^{2} = 0.3$. The indirect phenotypic variance $(\sigma_{{P_{\text{I}} }}^{2} )$ was set to $\frac{1}{3}\sigma_{{P_{\text{D}} }}^{2} = 0.33$ for all schemes with *d* = 0. With $d > 0$, depending on the average group size, values for $\upsigma_{{P_{\text{I}} }}^{2}$ differed (Table 3). Table 3 shows that when $d = 0$, $\sigma_{{P_{\text{I}} }}^{2}$ remains constant, whereas for $d > 0$, $\sigma_{{P_{\text{I}} }}^{2}$ decreased with group size. Thus, schemes are only comparable within each dilution parameter but schemes with different dilution parameters are not comparable. For each scheme, 50 replicates were simulated and, thus, the reported estimates were the average over 50 replicates.

### Group assignment

In the basic scenario, individuals were assigned randomly to groups. Thus, group mates were unrelated, except by chance, each family contributed individuals to many groups and each group contained members of multiple families. As an alternative, we also considered groups that were composed of members of two full-sib families, to investigate whether this improved the quality of the estimates (bias and precision), as was previously shown for the variance of IGE in schemes with constant group size [21, 22]. Distribution of the 8000 individuals from a family of size 40 across two-family groups was possible only for simulated schemes 14, 16 and 18 (Table 2). In these three schemes, each group consisted of members of two randomly selected full-sib families, each family contributing half of the group members, and each family contributing to several groups. For example, for scheme 14 with group sizes 6 and 10, the members from a given full-sib family of 40 individuals were allocated to five groups of size 6 (three members of the specific family per group) and to five groups of size 10 (five members of the specific family per group) (Additional file 2: Figure S1). However, for these three schemes, the number of groups shown in Table 2 for random designs and the number of groups for the two-family design do not match. Therefore, in order to make the comparison between the two-family design and the random design as fair as possible, they both consisted of 500 groups of a given size (i.e. 500 groups of 6 plus 500 groups of 10). The number of individuals (*T* = 8000) and families (full-sib family size of 40) were kept the same.

### Estimation of variance components

Genetic parameters (variance and covariance components) and the degree of dilution in the simulated data were estimated using the following mixed model [6]:

2 $${\mathbf{y}} = {\mathbf{Xb}} + {\mathbf{Z}}_{\text{D}} {\mathbf{a}}_{\text{D}} + {\mathbf{Z}}_{{{\text{I}}\left( {d,n} \right)}} {\mathbf{a}}_{{{\text{I}},\bar{n}}} + {\mathbf{E}}_{{{\text{I}}\left( {d,n} \right)}} {\mathbf{e}}_{{{\text{I}},\bar{n}}} + {\mathbf{e}} ,$$

where **y** is a vector of phenotypic records, **b** is a vector of the fixed effects of the two sexes, **X** is the design matrix corresponding to the fixed effect of sex, ${\mathbf{a}}_{\text{D}}$ is the vector of DGE, ${\mathbf{Z}}_{\text{D}}$ is the design matrix corresponding to DGE, ${\mathbf{a}}_{{{\text{I}},\bar{n}}}$ and ${\mathbf{e}}_{{{\text{I}},\bar{n}}}$ are vectors of IGE and indirect non-genetic effects, respectively, referring to the average group size, ${\mathbf{Z}}_{{{\text{I}}\left( {d,n} \right)}}$ and ${\mathbf{E}}_{{{\text{I}}\left( {d,n} \right)}}$ are design matrices corresponding to IGE and indirect non-genetic effects, respectively, which depend on the dilution parameter (*d*) and on group size (*n*), and ${\mathbf{e}}$ is a vector of residuals. Elements of ${\mathbf{Z}}_{{{\text{I}}\left( {d,n} \right)}}$ are [6]:

$${\mathbf{Z}}_{{{\text{I}}\left( {d,n} \right)}} \left( {i,j} \right) = \left( {\frac{{\bar{n} - 1}}{n - 1}} \right)^{d} \;{\text{when}}\;j\;{\text{was}}\;{\text{a}}\;{\text{groupmate}}\;{\text{of}}\;i ,$$

and $${\mathbf{Z}}_{{{\text{I}}\left( {d,n} \right)}} \left( {i,j} \right) = 0$$, otherwise.

Elements of ${\mathbf{E}}_{{{\text{I}}\left( {d,n} \right)}}$ were computed the same way as the elements of ${\mathbf{Z}}_{{{\text{I}}\left( {d,n} \right)}}$.

Direct $( {\mathbf{a}}_{\text{D}} )$ and indirect genetic effects $({\mathbf{a}}_{\text{I}} )$ were assumed to follow a bivariate normal distribution: $$\left[ {\begin{array}{*{20}c} {{\mathbf{a}}_{\text{D}} } \\ {{\mathbf{a}}_{\text{I}} } \\ \end{array} } \right] \sim BVN\left( {0,{\mathbf{C}} \otimes {\mathbf{A}}} \right)$$, where ${\mathbf{C}}$ is a 2*2 direct–indirect genetic (co)variance matrix $$\left[ {\begin{array}{*{20}c} {\sigma_{{a_{\text{D}} }}^{2} } & {\sigma_{{a_{\text{DI}} }} } \\ {\sigma_{{a_{\text{DI}} }} } & {\sigma_{{a_{\text{I}} }}^{2} } \\ \end{array} } \right]$$, and ${\mathbf{A}}$ is the additive genetic relationship matrix calculated from the pedigree. Residual effects were assumed to be normally distributed as: ${\mathbf{e}} \sim N\left( {0,{\mathbf{I}}\upsigma_{\text{e}} } \right)$.

Note that when group size is constant, fitting indirect non-genetic effects (the ${\mathbf{E}}_{{{\text{I}}\left( {d,n} \right)}} {\mathbf{e}}_{{{\text{I}},\bar{n}}}$ term) is equivalent to fitting a random group effect [24], but this is not the case when group size varies. Since our simulated data included different group sizes and due to the dependency of the group variance on group size in model (2) (see formula 9a in Bijma [6]), this can only be captured by including indirect non-genetic effects in the model. The above mixed model was fitted with the DMU software using REML [25].

### Estimation of the dilution of IGE

To estimate *d*, the likelihood was computed for a set of values of *d* to identify its maximum. Thus, for each replicate, the dilution of IGE was estimated for different values of *d*, in steps of 0.04. The intervals for *d* were sufficiently large to avoid choosing the best *d* at the border of the interval. In other words, when the best *d* was on the border of the interval, the interval was expanded. Then, the best value for *d* was chosen based on the maximum likelihood.

### Bias and precision of the estimated parameters

To assess whether the estimates of the (co)variance components and of *d* were biased, differences between the true simulated values and means of estimates across 50 replicates were evaluated. To measure the precision of the estimates of (co)variances and genetic correlations, their standard errors were used to calculate the 95% confidence interval (parameter ± SE*1.96 rather than ± SE such that the same measure of confidence intervals was used for all parameters, including *d*). The longer the length of the 95% confidence interval was, the lower was the precision of the estimates and vice versa. Since for *d*, the SE was not obtained directly from DMU, the 95% confidence intervals for *d* were obtained from log-likelihood values and a Chi square statistic test with one degree of freedom.

## Results

### Bias and precision of parameter estimates

Estimates of both *d* and (co)variances were unbiased, irrespective of the *CV* and average group size (Additional file 3: Figure S2, Additional file 4: Figure S3 and Additional file 5: Figure S4). For all schemes, the true values of the parameters were within ± 2 SE of the mean estimated values.

**Fig. 1:**
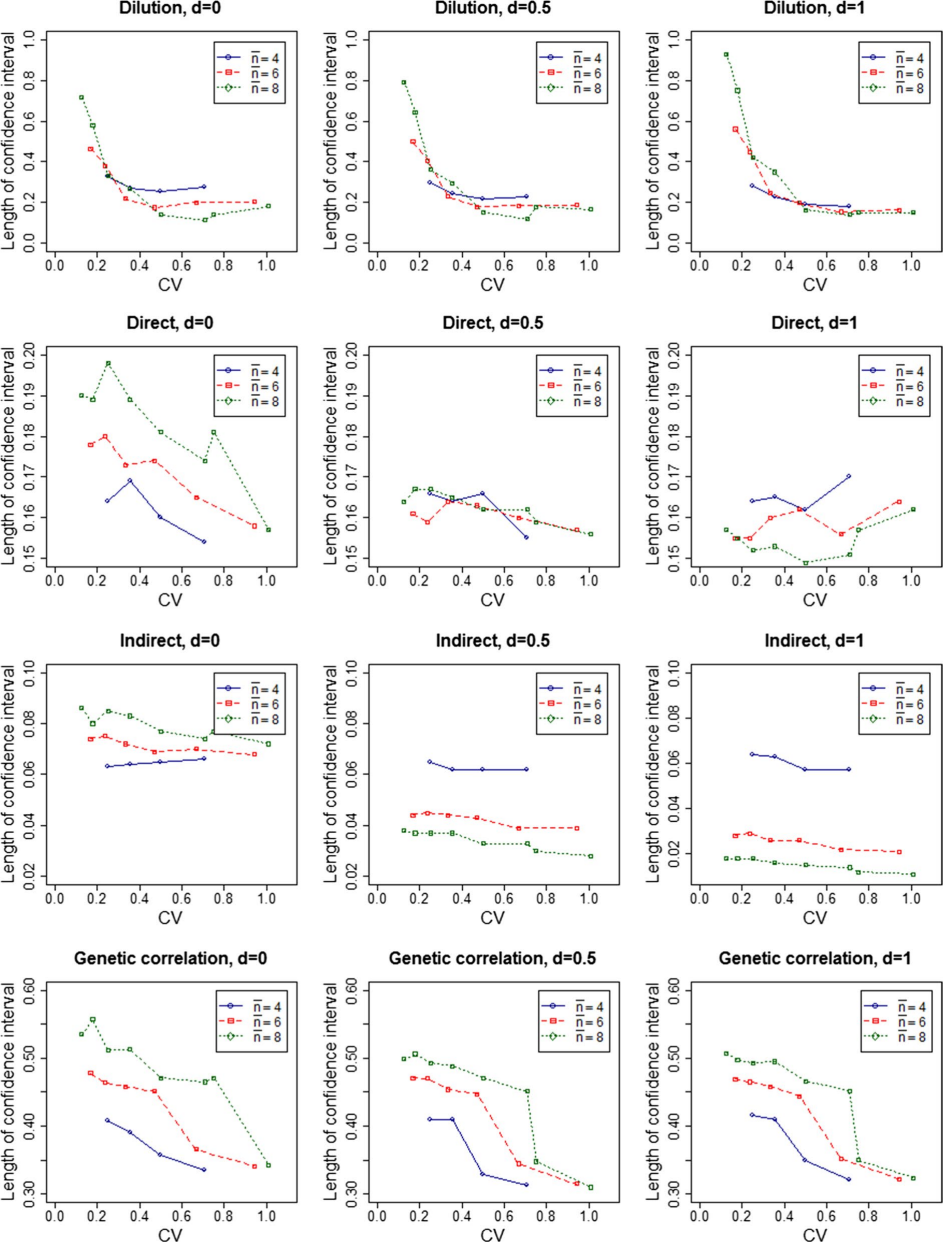
Length of confidence intervals for parameters for different group sizes. Lengths of the confidence intervals for dilution, variance of DGE, variance of IGE, and the genetic correlation between direct and indirect effects for different group sizes (simulated schemes) according to the *CV* of group size. Group compositions were random with respect to family

Figure 1 shows the lengths of the confidence intervals for all parameters (dilution, variances of DGE and IGE, and the genetic correlation between DGE and IGE) for different group sizes (schemes) as a function of the *CV* of group size. The schemes were compared within each *d* for three average group sizes (4, 6, and 8). Estimates, standard errors, and confidence intervals of *d* are in Additional file 1: Tables S1 and Additional file 6 Table S2. For all schemes, the length of the confidence intervals ranged from 0.114 to 0.927 for *d*, from 0.149 to 0.198 for the variance of DGE, from 0.011 to 0.086 for the variance of IGE, and from 0.310 to 0.557 for genetic correlation between DGE and IGE.

For all simulated *d* within each average group size, the length of the confidence interval for *d* decreased with increasing *CV* of group size, except for schemes 17 and 18 (2, 8, 14 vs. 2, 14) for which $\bar{n} = 8$ (Fig. 1) and Additional file 1: Table S1, for schemes 9 and 10 (2, 6, 10 vs. 2, 10) for which $\bar{n} = 6$, and for schemes 3 and 4 (2, 4, 6 vs. 2, 6) for which $\bar{n} = 4$ and *d* < 1. For these schemes, there was a slight increase in the length of the confidence interval for *d* as *CV* increased. For example, with *d* = 0 and $\bar{n} = 8$, the length of the confidence interval increased from 0.114 to 0.182 when *CV* increased from 0.707 to 1.010. To investigate whether this pattern is real or due to noise, the number of replicates was increased to 200 for these schemes, but the pattern remained the same. For the variance of DGE, we observed no clear pattern of the length of the confidence intervals with changes in the *CV* of group size. The precision of the estimate of the variance in IGE was expected to follow the same pattern as that for *d*, since these parameters are related and, indeed, in general the length of the confidence interval for the variance of IGE decreased as the *CV* increased (the same pattern as for *d*). For the genetic correlation between DGE and IGE, in general, a decrease in the length of the confidence interval with increasing *CV* of group size was also observed. However, some discrepancies in this pattern were found when the *CV* was smaller than 0.2.

### Random versus two-family design

We had expected that, for estimating *d*, the two-family design would perform better (shorter length of the confidence interval) than the random design but the two-family design was only slightly better (Additional file 7: Figure S5). The two-family design performed considerably better than the random design with respect to the precision of the estimate of the variance of IGE and of the genetic correlation, in agreement with results from previous studies with constant group size [21, 22]. For example, with the two-family design and *d* = 0, the length of the confidence interval for IGE was equal to 0.054 for scheme 14 (group sizes 6 and 10) and 0.055 for schemes 16 (group sizes 4 and 12) and 18 (group sizes 2 and 14), while the corresponding values for the random design were 0.085, 0.082, and 0.086 (Additional file 7: Figure S5).

For the variance of DGE, which design was better depended on *d*. For *d* = 0, the two-family design performed better than the random design, whereas for *d* > 0, the random design had a smaller confidence interval for the estimate of the variance of DGE (Additional file 7: Figure S5). When *d* = 0, superiority of the two-family design over the random design was largest for the scheme with the lower *CV* (scheme 6, 10 with *CV* = 0.353), whereas with *d* > 0, superiority of the random design was largest for the scheme with the highest *CV* (scheme 2, 14 with *CV* = 1.01).

## Discussion

In this study, we investigated whether dilution (*d*) can be estimated and whether this estimation depends on variation in group size (*CV*). Other relevant genetic parameters such as the variances of DGE and IGE and the genetic correlation between DGE and IGE were also estimated. Our findings show that *d* can be estimated unbiasedly with varying group size and that, in general, the precision of the estimate of *d* increases with increasing *CV* of group size. The group sizes used in this study for estimation of *d* ranged from 2 to 14, which applies for both chicken and pig breeding programs. However, we believe that our results on the estimability of dilution parameter also holds for group sizes larger than 14.

To our knowledge, the estimability of *d* and the bias and precision of its estimates have not been investigated to date. Some studies based on real data did investigate the dependency of IGE on group size and tested whether IGE become smaller when groups get larger (i.e. testing whether dilution exists) [10, 12, 16]. Some of these studies did in fact detect a dilution effect, while others did not. For example, Canario et al. [12] investigated the effect of group size (constant group sizes ranging from 5 to 15) on IGE for growth in pigs and found that both the indirect genetic and indirect litter effects decreased proportionally to group size. This means that the influences of pigs on the growth of their groupmates were diluted across more recipients in large groups compared to small groups. They compared several models with and without a dilution effect and models that took dilution into account increased the goodness of fit of the statistical model. Duijvesteijn et al. [16] investigated the dependence of IGE on group size for androstenon level in a population of boars (group sizes ranging from 3 to 11). They estimated the dilution for the IGE by computing the maximum likelihood of the model for *d* ranging from 0 to 1, with a step size of 0.25. Their results showed that the magnitude of IGE was not affected by group size, which they argued could be because of the relatively small group sizes they had. The degree of dilution also depends critically on the biological background of the trait [4, 6]. For a trait such as level of androstenon, which is a pheromone, dilution, is expected to be negligible because androstenon is spread by air in addition to being spread by physical contact [16]. In another study, Nielsen et al. [10] tested whether the IGE for growth (life time daily gain from birth to slaughter) in Danish pigs depended on group size [10]. Group sizes in their study ranged from 8 to 15. They found that IGE increased with increasing group size (i.e. they found that *d* was smaller than zero). Due to the imperfections of real data with varying group sizes, the studies that have investigated dilution are inconclusive. It is difficult to compare these studies because their power to estimate *d* is relatively low (e.g. the group sizes are sometimes different or the number of groups per group size is sometimes small). Therefore, before concluding that there is no dilution, it is necessary to be sure that it can be estimated. Our study shows that, given the mentioned designs and simulated schemes (group sizes) (see “Methods” section), it is in fact possible to estimate dilution.

Bijma [21] reported that more accurate parameter estimates of IGE were obtained with the two-family design than the random design with constant group size, and concluded that this design is optimal or near optimal for the estimation of the variance due to IGE. In our study, the two-family design was tested only for three simulated schemes with an average group size of 8 and the conclusion is that, in general, the two-family schemes performed better than random designs. However, differences between the two simulated schemes in terms of length of the confidence interval for estimates of *d* were small. This may be because family sizes and the number of groups were sufficiently large for estimation of *d* with a random design. For estimation of the variance of IGE and of the genetic correlation between DGE and IGE, superiority of the two-family design increased for $d = 0$, which is consistent with the results of [21]. For estimation of the variance of DGE, with $d > 0$, the random design performed better than the two-family design, because each family is distributed across a larger number of groups, making the random design more optimal for estimating DGE [22], since there is less confounding with IGE.

In addition to the nature of the trait of interest (when real data is used for dilution estimation), population structure, trait heritability (both direct and indirect heritability), genetic correlation between DGE and IGE, and group size may affect the estimation of dilution. In this study, data were simulated using a moderate indirect heritability $(h_{\text{I}}^{2} = 0.3)$ and a zero genetic correlation between DGE and IGE. When indirect heritability is low, the optimal family size and/or group size for precise estimation of dilution may be different. Generally, the lower the true heritability, the larger the optimal family size [26].

### Implications

Estimation of *d* is relevant when different group sizes are present in the data. Different group sizes can be relevant for breeding programs of several species for which animals are group-housed such as layers, pigs, and in aquaculture, where the group sizes vary due to mortality from diseases and involuntary culling. However, different group sizes are particularly relevant for pig breeding programs, in which each genetic line (breed) is typically represented on multiple farms that can have different group sizes, both between and within farms. In addition, in pig breeding programs, group sizes typically differ between the nucleus and commercial levels, with the larger group sizes at the commercial level.

Before implementing selection for social genetic effects in a breeding program, it is crucial to know whether or not dilution exists and to be able to estimate it. If, in reality, dilution existed but we did not or could not estimate it, response to selection (genetic progress that was created in the selection pure lines) could not be accurately predicted. For example, the prediction of the genetic progress which would be disseminated to the commercial animals would be inaccurate. In other words, ignoring dilution may result in reduced observed response to selection compared to the predicted response to selection, because an indirect genetic model without dilution may cause overestimation of the total heritable variance and response to selection in commercial animals, due to the improper interpretation of direct and indirect variances that contribute to the heritable variance in relation to group size. Therefore, to predict selection response at the commercial level as accurately as possible, estimation of the magnitude of dilution cannot be ignored.

## Conclusions

Dilution of indirect genetic effects could be detected in simulated data with varying group size and all parameters could be estimated without bias. The precision of the estimate of dilution was higher when the *CV* of group size was larger. For the estimation of dilution, schemes with groups composed of two families were slightly superior to the schemes with groups composed at random in terms of families.

## Additional files

**Additional file 1: Table S1.** Simulated and estimated parameters for the random designs.

**Additional file 2: Figure S1.** Graphical representation of the two-family group-making. Description: This figure shows how the groups for the two-family design (scheme 6, 10) were made. From each family with 40 full-sib offspring, 10 groups were made; five of the groups included 3 random full-sibs and the other five included 5 random full-sibs. To make the group size 6, the group size of 3 from one family were combined with the group size of 3 from another random family (e.g. here, family 1 and 2 contributed to a group size of 6, and family 2 and 3 contributed to another group size of 6). To make group size 10, the group size of 5 from one family were combined with the group size of 5 from another random family (e.g. here, family 1 and 2 contributed to a group size of 10, and family 1 and 3 contributed to another group size of 10). With 200 full-sib families, 500 groups of size equal to 6 and 500 groups size equal to 10 were made. Note that the similar pattern of two-family group-making was implemented for schemes 2, 14 and 4, 12.

**Additional file 3: Figure S2.** Description: Lower and upper confidence intervals for all parameters (dilution, variance of DGE, variance of IGE, and genetic correlation between direct and indirect effects) for different group sizes (schemes) with different *CV* and ${\bar{\text{n}}} = 4$. The black horizontal lines show the true simulated values and the black dots show the estimates. The group compositions are random.

**Additional file 4: Figure S3.** Lower and upper confidence intervals for all parameters (dilution, variance of DGE, variance of IGE, and genetic correlation between direct and indirect effects) for different group sizes (schemes) with different *CV* and ${\bar{\text{n}}} = 6$. The black horizontal lines show the true simulated values and the black dots show the estimates. The group compositions are random.

**Additional file 5: Figure S4.** Lower and upper confidence intervals for all parameters (dilution, variance of DGE, variance of IGE, and genetic correlation between direct and indirect effects) for different group sizes (schemes) with different *CV* and ${\bar{\text{n}}} = 8$. The black horizontal lines show the true simulated values and the black dots show the estimates. The group compositions are random.

**Additional file 6: Table S2.** Simulated and estimated parameters for the random design versus the two-family design when the number of groups was fixed (n_g_ = 500).

**Additional file 7: Figure S5.** Lengths of confidence intervals for two-family versus random schemes. Description: The lengths of confidence intervals for all parameters (dilution, variance of DGE, variance of IGE, and genetic correlation between direct and indirect effects) for schemes 2, 14; 4, 12; and 6, 10 (with $\bar{n} = 8$) where the random design was compared with the two-family design. The number of groups were fixed $(n_{g} = 500)$ for different group sizes.

## Data Availability

The datasets analyzed in this study were created by simulation and are available upon request.
