# Supplementary material for: Models with indirect genetic effects depending on group sizes: a simulation study assessing the precision of the estimates of the dilution parameter
Source: Genet Sel Evol. 2019 May 30;51:24. doi: 10.1186/s12711-019-0466-6 (PMC6543592; doi:10.1186/s12711-019-0466-6)
Supplement: Supplementary file 1 — Additional file 1: Table S1. Simulated and estimated parameters for the random designs. [file 12711_2019_466_MOESM1_ESM.docx]

| Scheme | n | $\sigma_{a_{D}}^{2}$ | $\sigma_{a_{I}}^{2}$ | $r_{a_{\mathrm{DI}}}$ |  | *d* | $\sigma_{a_{D}}^{2}(SE)$ | $\sigma_{a_{I}}^{2}(SE)$ | $r_{a_{\mathrm{DI}}}(SE)$ | *d* (upper,lower)* |
| --- | --- | --- | --- | --- | --- | --- | --- | --- | --- | --- |
| Simulated values | | | | | | | Estimated values | | | |
| Schemes with simulated *d*=0 | | | | | | | | | | |
| 1 | 3, 4, 5 | 0.3 | 0.1 | 0 |  | 0 | 0.300 (0.042) | 0.097 (0.016) | -0.001 (0.104) | 0.008 (-0.164,0.164) |
| 2 | 3, 5 | 0.3 | 0.1 | 0 |  | 0 | 0.316 (0.043) | 0.100 (0.016) | 0.009 (0.100) | 0.017 (-0.130,0.142) |
| 3 | 2, 4, 6 | 0.3 | 0.1 | 0 |  | 0 | 0.302 (0.041) | 0.098 (0.017) | -0.016 (0.091) | 0.010 (-0.119,0.134) |
| 4 | 2, 6 | 0.3 | 0.1 | 0 |  | 0 | 0.292 (0.039) | 0.099 (0.017) | -0.012 (0.085) | 0.030 (-0.110,0.163) |
| 5 | 5, 6, 7 | 0.3 | 0.1 | 0 |  | 0 | 0.300 (0.045) | 0.102 (0.019) | -0.013 (0.122) | 0.002 (-0.232,0.232) |
| 6 | 5, 7 | 0.3 | 0.1 | 0 |  | 0 | 0.306 (0.046) | 0.105 (0.019) | 0.014 (0.118) | -0.007 (-0.198,0.182) |
| 7 | 4, 6, 8 | 0.3 | 0.1 | 0 |  | 0 | 0.295 (0.044) | 0.102 (0.018) | -0.005 (0.117) | 0.001 (-0.114,0.106) |
| 8 | 4, 8 | 0.3 | 0.1 | 0 |  | 0 | 0.302 (0.044) | 0.096 (0.018) | -0.028 (0.115) | 0.026 (-0.066,0.110) |
| 9 | 2, 6, 10 | 0.3 | 0.1 | 0 |  | 0 | 0.307 (0.042) | 0.101 (0.018) | 0.007 (0.093) | 0.005 (-0.099,0.101) |
| 10 | 2, 10 | 0.3 | 0.1 | 0 |  | 0 | 0.303 (0.040) | 0.097 (0.017) | -0.014 (0.087) | 0.008 (-0.095,0.108) |
| 11 | 7, 8, 9 | 0.3 | 0.1 | 0 |  | 0 | 0.300 (0.048) | 0.105 (0.022) | -0.013 (0.136) | -0.007 (-0.367,0.346) |
| 12 | 7, 9 | 0.3 | 0.1 | 0 |  | 0 | 0.298 (0.048) | 0.093 (0.020) | -0.039 (0.142) | -0.054 (-0.346,0.235) |
| 13 | 6, 8, 10 | 0.3 | 0.1 | 0 |  | 0 | 0.323 (0.051) | 0.106 (0.022) | 0.026 (0.131) | -0.011 (-0.181,0.152) |
| 14 | 6, 10 | 0.3 | 0.1 | 0 |  | 0 | 0.306 (0.048) | 0.104 (0.021) | 0.001 (0.131) | -0.008 (-0.144,0.126) |
| 15 | 4, 8, 12 | 0.3 | 0.1 | 0 |  | 0 | 0.306 (0.046) | 0.101 (0.020) | 0.014 (0.120) | -0.006 (-0.077,0.063) |
| 16 | 4, 12 | 0.3 | 0.1 | 0 |  | 0 | 0.297 (0.044) | 0.100 (0.019) | -0.025 (0.119) | 0.002 (-0.058,0.056) |
| 17 | 2, 8, 14 | 0.3 | 0.1 | 0 |  | 0 | 0.288 (0.040) | 0.099 (0.018) | -0.016 (0.097) | 0.006 (-0.089,0.098) |
| 18 | 2, 14 | 0.3 | 0.1 | 0 |  | 0 | 0.298 (0.040) | 0.102 (0.018) | 0.005 (0.087) | 0.001 (-0.089,0.093) |
| Schemes with simulated *d*=0.5 | | | | | | | | | | |
| 1 | 3, 4, 5 | 0.3 | 0.1 | 0 |  | 0.5 | 0.303 (0.042) | 0.102 (0.016) | -0.006 (0.104) | 0.513 (0.364,0.660) |
| 2 | 3, 5 | 0.3 | 0.1 | 0 |  | 0.5 | 0.299 (0.042) | 0.097 (0.016) | -0.022 (0.105) | 0.499 (0.374,0.616) |
| 3 | 2, 4, 6 | 0.3 | 0.1 | 0 |  | 0.5 | 0.312 (0.042) | 0.101 (0.016) | -0.015 (0.084) | 0.515 (0.403,0.621) |
| 4 | 2, 6 | 0.3 | 0.1 | 0 |  | 0.5 | 0.288 (0.040) | 0.104 (0.016) | 0.014 (0.080) | 0.475 (0.361,0.591) |
| 5 | 5, 6, 7 | 0.3 | 0.06 | 0 |  | 0.5 | 0.291 (0.041) | 0.058 (0.011) | -0.025 (0.120) | 0.498 (0.249,0.748) |
| 6 | 5, 7 | 0.3 | 0.06 | 0 |  | 0.5 | 0.286 (0.040) | 0.059 (0.011) | -0.047 (0.120) | 0.475 (0.273,0.678) |
| 7 | 4, 6, 8 | 0.3 | 0.06 | 0 |  | 0.5 | 0.299 (0.042) | 0.059 (0.011) | 0.008 (0.116) | 0.493 (0.374,0.606) |
| 8 | 4, 8 | 0.3 | 0.06 | 0 |  | 0.5 | 0.298 (0.042) | 0.058 (0.011) | 0.015 (0.114) | 0.502 (0.410,0.590) |
| 9 | 2, 6, 10 | 0.3 | 0.06 | 0 |  | 0.5 | 0.305 (0.041) | 0.059 (0.010) | -0.007 (0.088) | 0.500 (0.403,0.589) |
| 10 | 2, 10 | 0.3 | 0.06 | 0 |  | 0.5 | 0.300 (0.040) | 0.062 (0.010) | 0.000 (0.080) | 0.486 (0.394,0.582) |
| 11 | 7, 8, 9 | 0.3 | 0.043 | 0 |  | 0.5 | 0.298 (0.042) | 0.044 (0.010) | 0.002 (0.127) | 0.553 (0.155,0.945) |
| 12 | 7, 9 | 0.3 | 0.043 | 0 |  | 0.5 | 0.306 (0.043) | 0.042 (0.099) | -0.005 (0.129) | 0.458 (0.137,0.780) |
| 13 | 6, 8, 10 | 0.3 | 0.043 | 0 |  | 0.5 | 0.307 (0.043) | 0.044 (0.099) | 0.011 (0.126) | 0.500 (0.325,0.686) |
| 14 | 6, 10 | 0.3 | 0.043 | 0 |  | 0.5 | 0.303 (0.042) | 0.044 (0.099) | 0.032 (0.124) | 0.515 (0.366,0.661) |
| 15 | 4, 8, 12 | 0.3 | 0.043 | 0 |  | 0.5 | 0.299 (0.041) | 0.041 (0.099) | -0.001 (0.120) | 0.492 (0.416,0.569) |
| 16 | 4, 12 | 0.3 | 0.043 | 0 |  | 0.5 | 0.300 (0.041) | 0.043 (0.008) | -0.005 (0.115) | 0.494 (0.430,0.550) |
| 17 | 2, 8, 14 | 0.3 | 0.043 | 0 |  | 0.5 | 0.308 (0.041) | 0.044 (0.008) | 0.004 (0.089) | 0.486 (0.393,0.573) |
| 18 | 2, 14 | 0.3 | 0.043 | 0 |  | 0.5 | 0.300 (0.040) | 0.044 (0.007) | -0.008 (0.079) | 0.509 (0.422,0.588) |
| Schemes with simulated *d*=1 | | | | | | | | | | |
| 1 | 3, 4, 5 | 0.3 | 0.1 | 0 |  | 1 | 0.296 (0.042) | 0.102 (0.016) | -0.013 (0.106) | 0.994 (0.857,1.137) |
| 2 | 3, 5 | 0.3 | 0.1 | 0 |  | 1 | 0.299 (0.042) | 0.102 (0.016) | 0.004 (0.104) | 0.994 (0.878,1.105) |
| 3 | 2, 4, 6 | 0.3 | 0.1 | 0 |  | 1 | 0.293 (0.041) | 0.097 (0.014) | 0.015 (0.089) | 0.998 (0.893,1.083) |
| 4 | 2, 6 | 0.3 | 0.1 | 0 |  | 1 | 0.310 (0.043) | 0.101 (0.015) | 0.003 (0.082) | 0.995 (0.894,1.072) |
| 5 | 5, 6, 7 | 0.3 | 0.036 | 0 |  | 1 | 0.295 (0.040) | 0.035 (0.007) | 0.013 (0.120) | 0.966 (0.684,1.245) |
| 6 | 5, 7 | 0.3 | 0.036 | 0 |  | 1 | 0.296 (0.040) | 0.036 (0.007) | -0.026 (0.119) | 0.999 (0.774,1.223) |
| 7 | 4, 6, 8 | 0.3 | 0.036 | 0 |  | 1 | 0.306 (0.041) | 0.034 (0.007) | -0.021 (0.117) | 1.014 (0.890,1.135) |
| 8 | 4, 8 | 0.3 | 0.036 | 0 |  | 1 | 0.311 (0.041) | 0.035 (0.007) | 0.012 (0.113) | 0.985 (0.888,1.086) |
| 9 | 2, 6, 10 | 0.3 | 0.036 | 0 |  | 1 | 0.296 (0.040) | 0.036 (0.006) | -0.010 (0.090) | 0.997 (0.910,1.065) |
| 10 | 2, 10 | 0.3 | 0.036 | 0 |  | 1 | 0.308 (0.042) | 0.036 (0.005) | -0.004 (0.082) | 0.993 (0.897,1.060) |
| 11 | 7, 8, 9 | 0.3 | 0.018 | 0 |  | 1 | 0.309 (0.040) | 0.019 (0.004) | 0.005 (0.129) | 0.946 (0.485,1.412) |
| 12 | 7, 9 | 0.3 | 0.018 | 0 |  | 1 | 0.304 (0.039) | 0.019 (0.005) | 0.026 (0.127) | 0.968 (0.590,1.341) |
| 13 | 6, 8, 10 | 0.3 | 0.018 | 0 |  | 1 | 0.297 (0.039) | 0.020 (0.005) | -0.019 (0.126) | 0.996 (0.784,1.207) |
| 14 | 6, 10 | 0.3 | 0.018 | 0 |  | 1 | 0.300 (0.039) | 0.018 (0.004) | 0.004 (0.126) | 1.022 (0.848,1.198) |
| 15 | 4, 8, 12 | 0.3 | 0.018 | 0 |  | 1 | 0.289 (0.038) | 0.018 (0.004) | -0.017 (0.119) | 1.007 (0.925,1.088) |
| 16 | 4, 12 | 0.3 | 0.018 | 0 |  | 1 | 0.288 (0.038) | 0.019 (0.004) | 0.003 (0.115) | 1.003 (0.934,1.074) |
| 17 | 2, 8, 14 | 0.3 | 0.018 | 0 |  | 1 | 0.305 (0.040) | 0.019 (0.003) | -0.013 (0.089) | 0.988 (0.899,1.050) |
| 18 | 2, 14 | 0.3 | 0.018 | 0 |  | 1 | 0.306 (0.041) | 0.019 (0.003) | 0.016 (0.083) | 0.984 (0.895,1.047) |

* Upper and lower bands for confidence interval. These values were obtained using the chi-square statistic test.
