# Supplementary material for: Models with indirect genetic effects depending on group sizes: a simulation study assessing the precision of the estimates of the dilution parameter
Source: Genet Sel Evol. 2019 May 30;51:24. doi: 10.1186/s12711-019-0466-6 (PMC6543592; doi:10.1186/s12711-019-0466-6)
Supplement: Supplementary file 2 — Additional file 2: Figure S1. Graphical representation of the two-family group-making. Description: This figure shows how the groups for the two-family design (scheme 6, 10) were made. From each family with 40 full-sib offspring, 10 groups were made; five of the groups included 3 random full-sibs and the other five included 5 random full-sibs. To make the group size 6, the group size of 3 from one family were combined with the group size of 3 from another random family (e.g. here, family 1 and 2 contributed to a group size of 6, and family 2 and 3 contributed to another group size of 6). To make group size 10, the group size of 5 from one family were combined with the group size of 5 from another random family (e.g. here, family 1 and 2 contributed to a group size of 10, and family 1 and 3 contributed to another group size of 10). With 200 full-sib families, 500 groups of size equal to 6 and 500 groups size equal to 10 were made. Note that the similar pattern of two-family group-making was implemented for schemes 2, 14 and 4, 12. [file 12711_2019_466_MOESM2_ESM.pptx]

## Slide 1
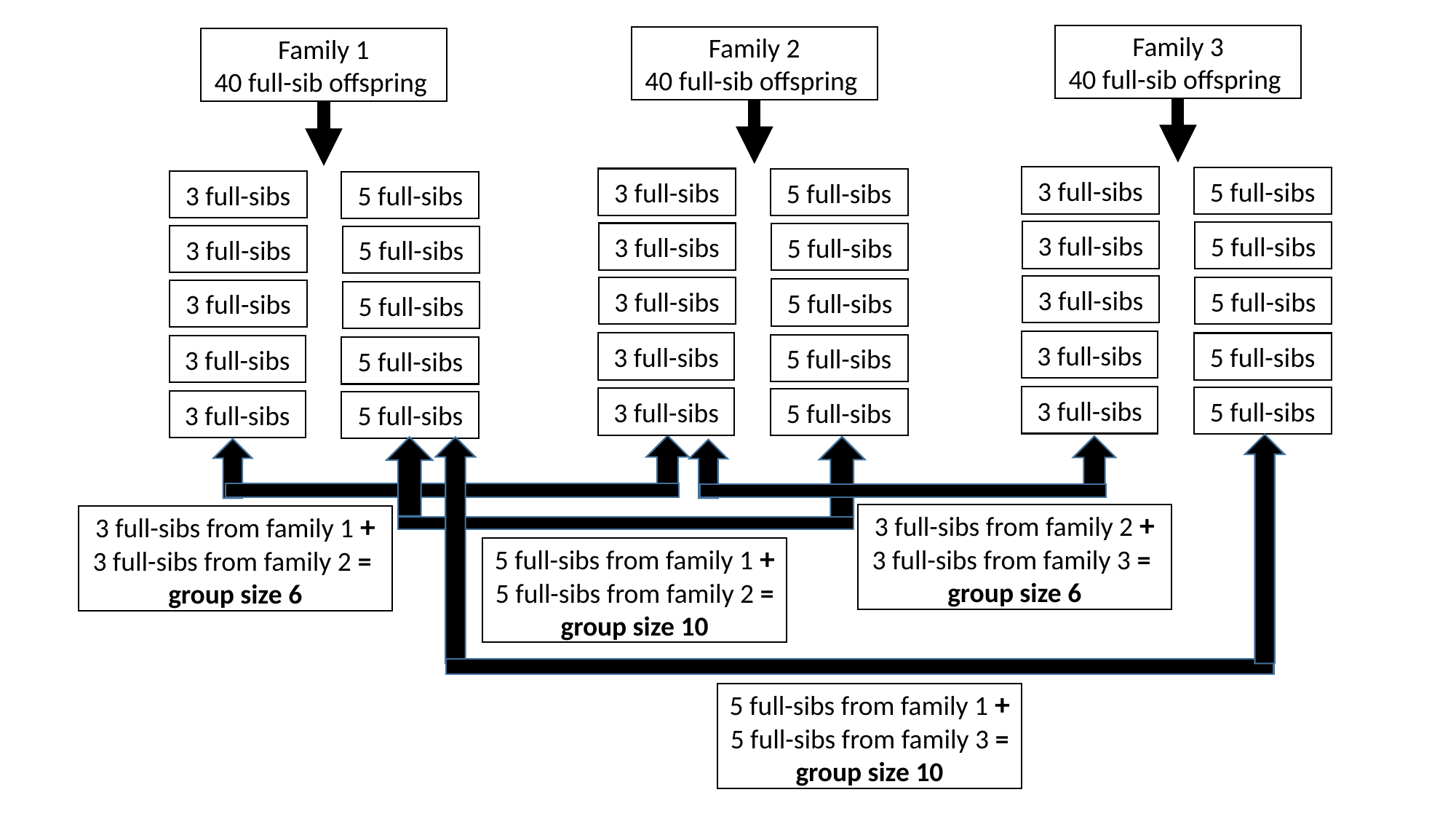

Family 3
40 full-sib offspring
Family 2
40 full-sib offspring
Family 1
40 full-sib offspring
3 full-sibs
5 full-sibs
3 full-sibs
5 full-sibs
3 full-sibs
5 full-sibs
3 full-sibs
5 full-sibs
3 full-sibs
5 full-sibs
3 full-sibs
5 full-sibs
3 full-sibs
3 full-sibs
5 full-sibs
5 full-sibs
3 full-sibs
5 full-sibs
3 full-sibs
3 full-sibs
5 full-sibs
5 full-sibs
3 full-sibs
5 full-sibs
3 full-sibs
5 full-sibs
3 full-sibs
5 full-sibs
3 full-sibs
5 full-sibs
3 full-sibs from family 2 +
3 full-sibs from family 3 =
group size 6
3 full-sibs from family 1 +
3 full-sibs from family 2 =
group size 6
5 full-sibs from family 1 +
5 full-sibs from family 2 =
group size 10
5 full-sibs from family 1 +
5 full-sibs from family 3 =
group size 10
