# Supplementary material for: Models with indirect genetic effects depending on group sizes: a simulation study assessing the precision of the estimates of the dilution parameter
Source: Genet Sel Evol. 2019 May 30;51:24. doi: 10.1186/s12711-019-0466-6 (PMC6543592; doi:10.1186/s12711-019-0466-6)
Supplement: Supplementary file 5 — Additional file 5: Figure S4. Lower and upper confidence intervals for all parameters (dilution, variance of DGE, variance of IGE, and genetic correlation between direct and indirect effects) for different group sizes (schemes) with different CV and ${\bar{\text{n}}} = 8$n¯=8. The black horizontal lines show the true simulated values and the black dots show the estimates. The group compositions are random. [file 12711_2019_466_MOESM5_ESM.pdf]

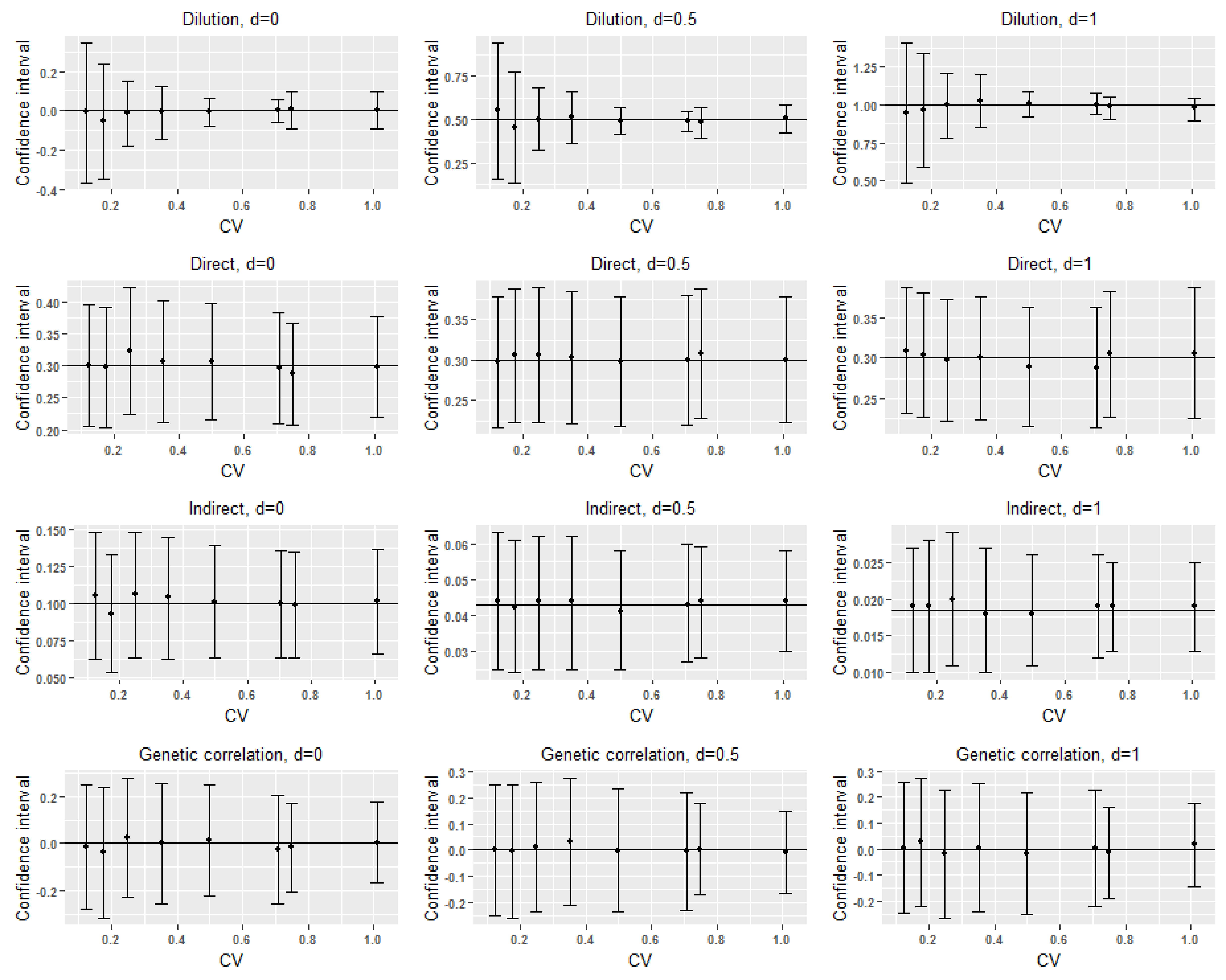

Dilution, d=0
Dilution, d=0.5
Dilution, d=1
Direct, d=0
Direct, d=0.5
Direct, d=1
Indirect, d=0
Indirect, d=0.5
Indirect, d=1
Genetic correlation, d=0
Genetic correlation, d=0.5
Genetic correlation, d=1
Confidence interval
CV
