# Supplementary material for: Models with indirect genetic effects depending on group sizes: a simulation study assessing the precision of the estimates of the dilution parameter
Source: Genet Sel Evol. 2019 May 30;51:24. doi: 10.1186/s12711-019-0466-6 (PMC6543592; doi:10.1186/s12711-019-0466-6)
Supplement: Supplementary file 6 — Additional file 6: Table S2. Simulated and estimated parameters for the random design versus the two-family design when the number of groups was fixed (ng = 500). [file 12711_2019_466_MOESM6_ESM.docx]

| Scheme | n | $\sigma_{a_{D}}^{2}$ | $\sigma_{a_{I}}^{2}$ | $r_{a_{\mathrm{DI}}}$ | | *d* | $\sigma_{a_{D}}^{2}(SE)$ | $\sigma_{a_{I}}^{2}(SE)$ | $r_{a_{\mathrm{DI}}}(SE)$ | *d* (upper,lower)* |
| --- | --- | --- | --- | --- | --- | --- | --- | --- | --- | --- |
| Simulated values | | | | | | | Estimated values | | | |
| Schemes with simulated *d*=0 | | | | | | | | | | |
| Family^1^ | 6, 10 | 0.3 | 0.1 | 0 | 0 | | 0.292 (0.045) | 0.099 (0.014) | -0.025 (0.105) | 0.021 (-0.102,0.142) |
| Rand^2^ | 6, 10 | 0.3 | 0.1 | 0 | 0 | | 0.294 (0.048) | 0.102 (0.022) | -0.016 (0.137) | 0.030 (-0.110,0.162) |
| Family | 4, 12 | 0.3 | 0.1 | 0 | 0 | | 0.307 (0.047) | 0.100 (0.014) | -0.004 (0.102) | 0.000 (-0.058,0.059) |
| Rand | 4, 12 | 0.3 | 0.1 | 0 | 0 | | 0.302 (0.047) | 0.098 (0.021) | -0.017 (0.131) | -0.003 (-0.074,0.060) |
| Family | 2, 14 | 0.3 | 0.1 | 0 | 0 | | 0.293 (0.045) | 0.097 (0.014) | -0.009 (0.101) | -0.028 (-0.180,0.080) |
| Rand | 2, 14 | 0.3 | 0.1 | 0 | 0 | | 0.304 (0.047) | 0.096 (0.022) | -0.029 (0.127) | -0.009 (-0.244,0.139) |
| Schemes with simulated *d*=0.5 | | | | | | | | | | |
| Family | 6, 10 | 0.3 | 0.043 | 0 | 0.5 | | 0.304 (0.044) | 0.042 (0.006) | -0.003 (0.102) | 0.509 (0.370,0.643) |
| Rand | 6, 10 | 0.3 | 0.043 | 0 | 0.5 | | 0.309 (0.043) | 0.046 (0.010) | 0.007 (0.126) | 0.519 (0.367,0.668) |
| Family | 4, 12 | 0.3 | 0.043 | 0 | 0.5 | | 0.298 (0.043) | 0.044 (0.006) | -0.003 (0.101) | 0.510 (0.451,0.566) |
| Rand | 4, 12 | 0.3 | 0.043 | 0 | 0.5 | | 0.296 (0.041) | 0.044 (0.010) | 0.026 (0.124) | 0.494 (0.427,0.559) |
| Family | 2, 14 | 0.3 | 0.043 | 0 | 0.5 | | 0.300 (0.043) | 0.043 (0.006) | 0.012 (0.096) | 0.498 (0.399,0.565) |
| Rand | 2, 14 | 0.3 | 0.043 | 0 | 0.5 | | 0.296 (0.039) | 0.044 (0.010) | 0.003 (0.113) | 0.493 (0.340,0.588) |
| Schemes with simulated *d*=1 | | | | | | | | | | |
| Family | 6, 10 | 0.3 | 0.018 | 0 | 1 | | 0.293 (0.041) | 0.018 (0.003) | -0.013 (0.104) | 1.010 (0.854,1.167) |
| Rand | 6, 10 | 0.3 | 0.018 | 0 | 1 | | 0.304 (0.039) | 0.018 (0.004) | 0.006 (0.130) | 0.985 (0.804,1.161) |
| Family | 4, 12 | 0.3 | 0.018 | 0 | 1 | | 0.308 (0.043) | 0.018 (0.003) | -0.012 (0.104) | 1.006 (0.942,1.070) |
| Rand | 4, 12 | 0.3 | 0.018 | 0 | 1 | | 0.300 (0.039) | 0.018 (0.004) | -0.013 (0.124) | 1.013 (0.936,1.085) |
| Family | 2, 14 | 0.3 | 0.018 | 0 | 1 | | 0.299 (0.042) | 0.018 (0.003) | 0.008 (0.099) | 0.986 (0.905,1.039) |
| Rand | 2, 14 | 0.3 | 0.018 | 0 | 1 | | 0.291 (0.037) | 0.019 (0.004) | -0.019 (0.111) | 0.978 (0.862,1.045) |

* Upper and lower bands for confidence interval. These values were obtained using the chi-square statistic test.

^1^ Two-family design.

^2^ Random design.
