# Supplementary material for: Models with indirect genetic effects depending on group sizes: a simulation study assessing the precision of the estimates of the dilution parameter
Source: Genet Sel Evol. 2019 May 30;51:24. doi: 10.1186/s12711-019-0466-6 (PMC6543592; doi:10.1186/s12711-019-0466-6)
Supplement: Supplementary file 7 — Additional file 7: Figure S5. Lengths of confidence intervals for two-family versus random schemes. Description: The lengths of confidence intervals for all parameters (dilution, variance of DGE, variance of IGE, and genetic correlation between direct and indirect effects) for schemes 2, 14; 4, 12; and 6, 10 (with $\bar{n} = 8$n¯=8) where the random design was compared with the two-family design. The number of groups were fixed $(n_{g} = 500)$(ng=500) for different group sizes. [file 12711_2019_466_MOESM7_ESM.pdf]

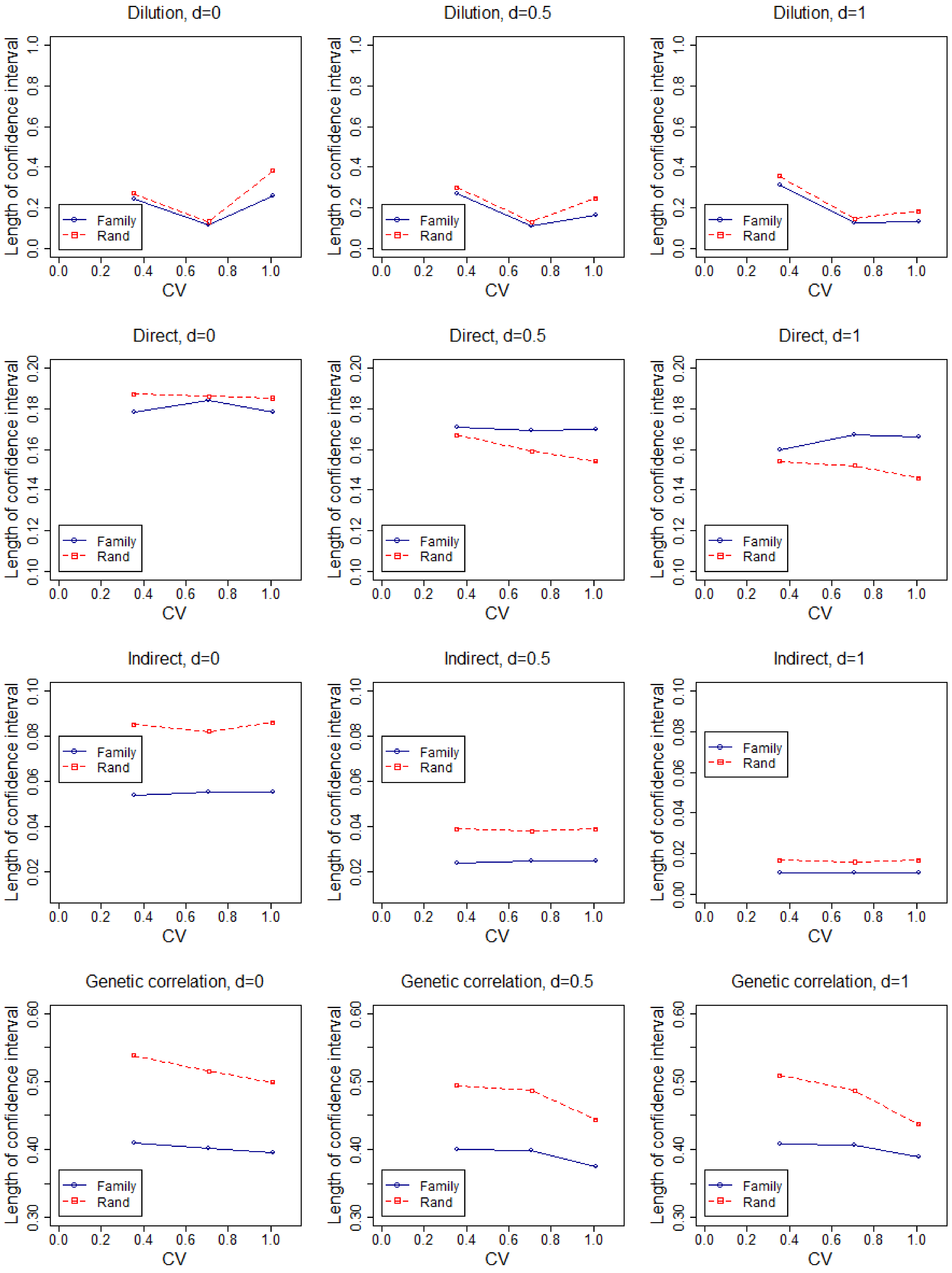

Dilution, d=0
Dilution, d=0.5
Dilution, d=1
Direct, d=0
Direct, d=0.5
Direct, d=1
Indirect, d=0
Indirect, d=0.5
Indirect, d=1
Genetic correlation, d=0
Genetic correlation, d=0.5
Genetic correlation, d=1
Length of confidence interval
CV
Family
Rand
